# Predictors of the resumption of menses in adolescent anorexia nervosa

**DOI:** 10.1186/1471-244X-13-308

**Published:** 2013-11-15

**Authors:** Astrid Dempfle, Beate Herpertz-Dahlmann, Nina Timmesfeld, Reinhild Schwarte, Karin M Egberts, Ernst Pfeiffer, Christian Fleischhaker, Christoph Wewetzer, Katharina Bühren

**Affiliations:** 1 Institute of Medical Biometry and Epidemiology, Philipps-University Marburg, Marburg, Germany; 2 Department of Child and Adolescent Psychiatry, Psychosomatics and Psychotherapy, University Hospital of the RWTH Aachen, Aachen, Germany; 3 Department of Child and Adolescent Psychiatry, Psychosomatics and Psychotherapy, University Hospital Würzburg, Würzburg, Germany; 4 Department of Child and Adolescent Psychiatry, Psychosomatics and Psychotherapy, Charité Universitätsmedizin Berlin, Berlin, Germany; 5 Department of Child and Adolescent Psychiatry and Psychotherapy, University Medical Center Freiburg, Freiburg, Germany; 6 Department of Child and Adolescent Psychiatry and Psychotherapy, Kliniken der Stadt Köln, Köln, Germany

**Keywords:** Resumption of menses, Adolescence anorexia nervosa, Target weight, Menstrual recovery, Outcome, Body mass index, Menarche, Amenorrhea

## Abstract

**Background:**

The resumption of menses is an important indicator of recovery in anorexia nervosa (AN). Patients with early-onset AN are at particularly great risk of suffering from the long-term physical and psychological consequences of persistent gonadal dysfunction. However, the clinical variables that predict the recovery of menstrual function during weight gain in AN remain poorly understood. The aim of this study was to investigate the impact of several clinical parameters on the resumption of menses in first-onset adolescent AN in a large, well-characterized, homogenous sample that was followed-up for 12 months.

**Methods:**

A total of 172 female adolescent patients with first-onset AN according to DSM-IV criteria were recruited for inclusion in a randomized, multi-center, German clinical trial. Menstrual status and clinical variables (i.e., premorbid body mass index (BMI), age at onset, duration of illness, duration of hospital treatment, achievement of target weight at discharge, and BMI) were assessed at the time of admission to or discharge from hospital treatment and at a 12-month follow-up. Based on German reference data, we calculated the percentage of expected body weight (%EBW), BMI percentile, and BMI standard deviation score (BMI-SDS) for all time points to investigate the relationship between different weight measurements and resumption of menses.

**Results:**

Forty-seven percent of the patients spontaneously began menstruating during the follow-up period. %EBW at the 12-month follow-up was strongly correlated with the resumption of menses. The absence of menarche before admission, a higher premorbid BMI, discharge below target weight, and a longer duration of hospital treatment were the most relevant prognostic factors for continued amenorrhea.

**Conclusions:**

The recovery of menstrual function in adolescent patients with AN should be a major treatment goal to prevent severe long-term physical and psychological sequelae. Patients with premenarchal onset of AN are at particular risk for protracted amenorrhea despite weight rehabilitation. Reaching and maintaining a target weight between the 15^th^ and 20^th^ BMI percentile is favorable for the resumption of menses within 12 months. Whether patients with a higher premorbid BMI may benefit from a higher target weight needs to be investigated in further studies.

## Background

Anorexia nervosa (AN) is a psychiatric disorder that typically first occurs in adolescence and is the third-most common chronic illness in this age group, after asthma and obesity [1]. AN often has a protracted course and is associated with an array of medical and psychiatric comorbidities [2,3].

Although the DSM-5 has abandoned amenorrhea as a diagnostic criterion for AN, there is general agreement that the resumption of menses is an important indicator of recovery from AN [4-6]. Thus, pediatric and psychiatric guidelines highlight the resumption of menses as an important goal in their recommendations for the treatment of AN [7-9].

Prolonged amenorrhea is a factor that likely contributes to poor physical outcome in AN. Patients with adolescent-onset AN are at particular risk of suffering from long-term physical and neuropsychiatric consequences of continuous gonadal dysfunction. Bone mineral accrual occurs mainly during adolescence [10]. Persistent amenorrhea at this age may lead to reduced peak bone mass and result in an increased risk for bone fractures later in life [11]. Studies have revealed that the normalization of body weight alone does not improve bone density; rather, the optimization of body weight and the normalization of gonadal status are necessary [12-14]. Moreover, in adolescent AN, starvation-induced hormonal deficiencies may lead to impairments in the maturation of certain brain regions that result in deficits in neuropsychological functioning [15-17]. Accordingly, it was shown that abnormal menstrual function in adolescent patients with AN is associated with deficits in verbal learning and memory, visual reproduction, working memory, and mathematical skills [15].

Weight rehabilitation is a prerequisite for reestablishing endocrine function [11,18,19]. During weight gain, fat mass increases resulting in the normalization of gonadal hormones and leptin levels. Leptin is a neuropeptide that is involved in various neuroendocrine and behavioral alterations associated with starvation [11,20]. However, some individuals remain amenorrheic for years despite stable weight recovery [21,22]. Currently, the clinical parameters that predict recovery of hypothalamic-pituitary-ovarian function during weight gain in AN remain poorly understood.

The ability to predict the magnitude of weight gain required for regular ovulatory cycles and a better understanding of the factors underlying the time lag between weight recovery and menstrual resumption would aid clinicians in determining their patients’ target weights. BMI at admission [23], BMI at discharge [24], and BMI before the onset of AN (“premorbid BMI”) [25,26] have been found to be important factors associated with the recovery of menstrual function and thus may aid in predicting the probability of resumption of menses in patients with AN.

However, there is no agreement on the definition of target weight. The American Psychiatric Association states that “a healthy goal weight for female patients is the weight at which normal menstruation and ovulation are restored” and that a healthy weight is “approximately the same weight at which full physical and psychological vigor were present” [7]. These definitions are based on the finding of two studies [27,28] that normal menstrual cycles are more likely to occur if the difference between the target weight and the body weight at which menstruation ceased is small.

Further studies have shown that in adult women with AN, a BMI higher than 18 kg/m^2^ is a relevant factor associated with the normalization of reproductive function [24-26,29].

In adolescents, the determination of target weight is even more complex, as both height and weight are expected to increase during this developmental period [30] and absolute BMI changes with age [31]. Golden et al. [30] recommended the use of BMI percentile ranges of the 14-39^th^ percentile to assign a treatment goal weight. Other researchers calculated the percent of expected body weight (%EBW) on the basis of BMI percentiles according to the recommendation of Le Grange et al. [32] and found that 2/3 of the patients resume menses at 95% of their expected body weight (%EBW) [33]. Swenne et al. [28] suggested the use of BMI standard deviation scores (BMI-SDS) to determine weight requirements for the resumption of menses in adolescents. Neither the American Psychiatric Association or American pediatric guidelines [9] nor the NICE guidelines provide a clear recommendation for the determination of target weight. The American Psychiatric Association notes that “the closer a patient is to his or her healthy body weight before discharge, the less the risk he or she has of relapsing and being readmitted” and recommends the use of population reference data (e.g., growth charts of the Centers for Disease Control and Prevention) to set individually appropriate and realistic goals for weight in adolescent patients [7,34]. In the German guidelines [8], the target weight in adolescents is defined as the 25^th^, but at least the 10^th^, BMI percentile, based on German reference data [35]. Thus, in our treatment program, we determined a discharge weight that was close to the target weight as defined above according to the German guidelines, and patients had to maintain this weight during outpatient treatment.

The aim of this study was to investigate the impact of several clinical parameters on the resumption of menses in a large, well-characterized, homogenous sample of first-onset adolescent AN patients who were followed up for 12 months.

## Methods

### Patients

The patients in this study were recruited within a randomized, multi-center clinical trial comparing inpatient and day-patient treatment for adolescent AN (the results of that investigation will be published elsewhere; [36]). The study was performed at four university hospitals (RWTH Aachen, University of Würzburg, Charité Universitätsmedizin Berlin, University of Freiburg and Medical Faculty of Mannheim of Heidelberg University) and one major general hospital (Köln) in various regions of Germany between February 2007 and April 2010, and the follow-up period lasted until April 2011. The inclusion criteria were as follows: female gender, age between 11 and 18 years, a diagnosis of AN based on DSM-IV criteria, and first admission for AN. The weight threshold for inclusion in the study was a BMI below the 10th percentile (based on a large German reference set [35]). This threshold is considered to be consistent with the international convention for the definition of AN [31,37] and corresponds to the German guidelines for eating disorders [8]. Patients were excluded from the study if they had an organic brain disease, a psychotic disorder, bipolar disorder, substance dependence or abuse, serious self-injurious behavior, or an IQ below 85. All patients who were admitted for inpatient treatment were screened for the study. The trial aimed to include a series of consecutively presenting adolescent patients with AN that was as complete as possible.

The study was approved by the local research ethics committees of the four participating universities (RWTH Aachen, University of Wuerzburg, Charité Universitaetsmedizin Berlin, University of Freiburg, Medical Faculty of Mannheim of Heidelberg University) and the ethics committee of the regional Medical Association (Nordrhein; Koeln-Holweide). After a complete description of the study was provided to the subjects, written informed consent was obtained from all participants and their parents or legal guardians.

All patients fulfilling the study criteria were initially admitted to a three-week inpatient care program. Subsequently, the patients were randomized into two treatment settings (continued inpatient or day-patient treatment). An identical multimodal multidisciplinary treatment program was used in both treatment arms that included weight restoration, individual and group nutritional counseling, cognitive-behavioral individual and group therapy, individual family sessions, and a group psychoeducation program for parents (for further details, see [38]). According to the German guidelines for eating disorders [8] and consistent with other clinical practices [30], patients were regularly discharged when they had maintained their predetermined target weight corresponding to the 15-20^th^ age-adjusted BMI percentile for two weeks. Discharge against medical advice was defined as premature drop-out from treatment without achievement of the predetermined target weight. After discharge, patients were provided access to the same outpatient treatment program, including cognitive-behavioral individual and group sessions, joint family sessions, and nutritional therapy (for further details, see [38]). A hospital readmission was initiated if the patients fell below a pre-determined weight (usually corresponding to the 10^th^ BMI percentile). The 12-month follow-up (12 months after admission) was defined as the primary outcome assessment time point.

### Menstrual status

Menstrual status was assessed at admission, discharge, and 12 months after admission. Brief assessments of menstrual status were also performed at regular outpatient visits. For amenorrheic patients, we assessed whether the patients were premenarchal (no menarche at age <16 years), had primary amenorrhea (no menarche by the age of ≥16 years [39]), or had secondary amenorrhea.

We compared two different patient groups: those who resumed menstruation spontaneously within the first 12 months after admission to the hospital treatment program and those who did not. The “menstruating group” consisted of patients who were menstruating at any time prior to follow-up and were not taking oral contraceptives (OCs) or who reported spontaneous resumption of menses before the use of OCs. In the “amenorrheic group”, we included patients who were still amenorrheic at the 12-month follow-up. Patients were excluded if menstrual status information was missing at the 12-month follow-up or before the initiation of OCs.

### Clinical variables

Clinical variables were assessed at admission, discharge, and 12 months after admission. At each time point, the evaluations consisted of measurements of weight (measured with the patients in their underwear) and height, from which BMIs were calculated. Age- and sex-adjusted BMI standard deviation scores (BMI-SDS), BMI percentiles, and percentages of expected body weight (%EBW, where the expected body weight (EBW) is the median age-adjusted BMI (50th BMI-percentile), and, thus, the %EBW is the observed BMI/EBW × 100 [32]) were calculated based on a large German population-based normative data set [35]. This reference population for children and adolescents was compiled from measurements from 17 studies conducted in different regions in Germany between 1985 and 1999 (17147 males, 17274 females, 0 to 18 years of age). At admission, age, age at AN onset (defined as the earliest of the following times: (1) the approximate time at which weight loss first occurred; (2) at which, despite age-appropriate growth spurts, no further weight gain took place; or (3) at which secondary amenorrhea first occurred), duration of illness (the time between the onset of AN and admission), and premorbid BMI (BMI before illness onset) were assessed. At discharge, the duration of hospital treatment and discharge modality (regular discharge at target weight or discharge against medical advice based on persisting AN symptoms and failure to meet the target weight) were recorded for each patient.

### Statistical analysis

Patients who were excluded from the analysis of menstrual status and those who were included were compared with respect to all clinical variables available until discharge (see above) using ANOVA. To compare the menstruating and amenorrheic groups, we performed logistic regression analyses. The independent clinical variables available until discharge were used as predictors (quantitative variables: age at admission, age at AN onset, age at menarche, premorbid BMI and BMI, BMI-SDS, BMI percentile and %EBW (at admission and discharge), duration of hospital treatment; binary variables: premenarchal or postmenarchal state at admission, regular discharge or discharge against medical advice). To analyze the relationship between menstrual status and cuurent weight, we also used %EBW at the 12-month follow-up as an independent variable. Descriptive data (evaluated by univariate analyses) are provided separately for the two groups as the means and standard deviations (or numbers and percent), together with the p-values from univariate logistic regression. Multivariate model selection using a stepwise procedure based on the Akaike information criterion (AIC) was performed. The primary statistical analyses were performed using %EBW as an appropriate measure for body weight in adolescents; in addition, we also report absolute BMI (enabling a better comparison with studies in adult AN patients), BMI percentiles, and BMI-SDS.

To evaluate the predictive accuracy of the logistic models, we used the area under the receiver operating characteristic curve (AUC of the ROC; sensitivity vs. specificity). The AUC is the probability that the risk score of a randomly picked non-menstruating patient is less than the risk score of a menstruating patient and measures the discrimination ability of the variables included in the logistic model. We compared the AUCs of different ROC curves (from models with and without %EBW at follow-up) using DeLong’s test [40].

Treatment setting (inpatient or day-patient) was not considered in the present analysis of menstruation because the durations of treatment and therapy outcomes with respect to BMI at discharge and at the 12-month follow-up were similar in the two treatment arms (95% confidence interval for difference in BMI at 12-month follow-up: -0.11 to 1.02; [36]).

## Results

### Data at baseline and at the 12-month follow-up

A total of 172 patients were included in the clinical trial. At admission, all patients were amenorrheic. Most of the patients reported secondary amenorrhea, while 36 patients (20.9%) were premenarchal (31 patients) or had primary amenorrhea (5 patients).

At the 12-month follow-up, information about menstruation was missing in 13 patients, and 7 patients were taking OCs and had missing information regarding menstrual status prior to OC use. Thus, a total of 20 patients could not be included in our analyses. However, the excluded patients were similar to the included patients with respect to all investigated variables assessed until discharge (all p-values >0.15).

Therefore, the final sample used in the analysis included 152 patients. Seventy-two of these patients (47%) had spontaneously resumed menses during the follow-up period. However, 80 patients (53%) were still amenorrheic. Among these patients, 25 were premenarchal and 5 had primary amenorrhea. The clinical data at admission, discharge, and the 12-month follow-up are provided in Table 1.

**Table 1:** Clinical data at admission, discharge and 12 months after admission for patients with and without spontaneous resumption of menses within the first 12 months after admission

	**Menstruating**	**Amenorrheic**	**p-value**^**a**^
	**n = 72**	**n = 80**	
	**Mean (SD) or n (%)**	**Mean (SD) or n (%)**	
premorbid BMI (kg/m^2^)	20.2 (2.8)	19.9 (2.8)	0.61
premorbid BMI-percentile	49.0 (29.3)	49.4 (28.2)	0.94
premorbid BMI-SDS	0.0 (1.0)	0.0 (0.9)	0.96
premorbid %EBW	101.6 (14.0)	101.7 (13.8)	0.98
age at menarche (years)	12.5 (1.2)	12.3 (1.0)	0.49
age at onset of AN (years)	14.5 (1.4)	14.1 (1.4)	0.09
duration of illness (months)	11.5 (9.4)	9.9 (7.2)	0.23
no menarche at admission	3 (4.2%)	30 (37.5%)	<0.0001
- premenarchal	3 (4.2%)	25 (31.25%)	
- primary amenorrhea	0	5 (6.25%)	
age at admission (years)	15.4 (1.3)	14.9 (1.6)	0.03
BMI at admission (kg/m^2^)	15.3 (1.2)	14.8 (1.4)	0.02
BMI-percentile at admission	2.0 (3.1)	2.2 (5.1)	0.84
BMI-SDS at admission	′-2.0 (3.1)	-2.2 (5.1)	0.84
%EBW at admission	75.4 (6.1)	74.1 (7.1)	0.24
duration of treatment (weeks)	14.7 (5.4)	16.8 (7.2)	0.05
regular discharge*	56 (77.8%)	49 (61.2%)	0.03
BMI at discharge (kg/m^2^)	18.2 (0.9)	17.8 (1.1)	0.01
BMI-percentile at discharge	19.4 (8.0)	17.8 (1.1)	0.01
BMI-SDS at discharge	′-0.9 (0.5)	′-1.0 (0.4)	0.28
%EBW at discharge	89.2 (4.2)	88.4 (4.2)	0.21
BMI at 12-month follow-up (kg/m^2^)	18.8 (1.7)	17.5 (1.4)	<0.0001
BMI-percentile at 12-month follow-up	24.0 (21.3)	13.3 (14.1)	0.0013
BMI-SDS at 12-month follow-up	′-0.8 (0.7)	′-1.4 (0.7)	<0.0001
%EBW at 12-month follow-up	91.1 (8.7)	85.7 (7.0)	0.0003

BMI = body mass index, %EBW = % expected body weight.

*regular discharge = when target weight (between the 15 and 20th BMI percentiles) was maintained for two weeks.

^a^p-value from univariate logistic regression.

There was a strong association between patients’ %EBW at the 12-month follow-up and the resumption of menses (p = 0.0003, univariate logistic regression). The %EBW at follow-up was the variable that best discriminated between groups, as shown by an AUC of 0.69. Considering the absolute BMI data, this result means that a reduction in BMI of 1 kg/m^2^ approximately doubled the odds of persistent amenorrhea (OR = 1.94).

### Prognostic variables for the resumption of menstruation

**Figure 1:**
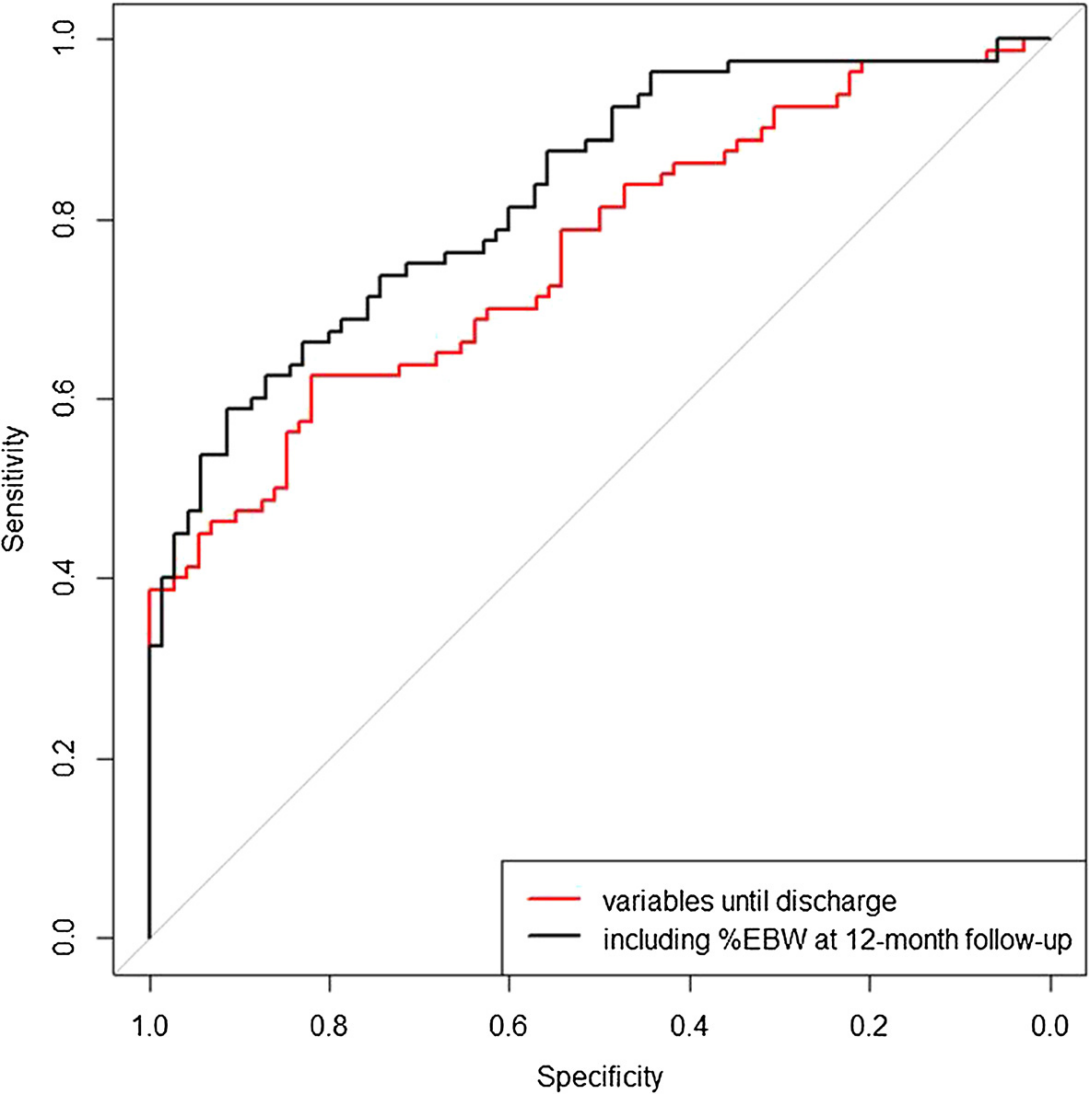
ROC of two different logistic regression models (red: model including premorbid %EBW, no menarche at admission, duration of hospital treatment and regular discharge with target weight; black: model including premorbid %EBW, no menarche at admission, duration of hospital treatment, regular discharge with target weight and %EBW at 12-month follow-up).

Among the variables that were available until discharge and could thus be used as prognostic factors, the most important variable for the resumption of menstruation was “no menarche at admission” (p = 0.00003, univariate logistic regression; AUC of 0.67). Patients who had never menstruated before had a 14-fold increase in the odds of remaining amenorrheic compared with the patients with secondary amenorrhea (OR = 13.8). A multiple logistic regression (adjusted for age; p = 0.17) revealed that no menarche (p = 0.0003), premorbid %EBW (p = 0.04), duration of hospital treatment (p = 0.03), and regular discharge at the target weight (p = 0.01) were the relevant prognostic factors prior to discharge. The joint predictive value of these clinical variables was high (AUC of 0.76). Although %EBW at the 12-month follow-up was strongly correlated with the resumption of menses, it did not add much to the discrimination power of the multivariate logistic regression (AUC of 0.83, p = 0.2 for the difference between AUCs, DeLong test, Figure 1). In the multiple logistic regression model, only no menarche at admission (p = 0.0005), premorbid %EBW (p = 0.007), and %EBW at follow-up (p = 0.0009) were significant, and the discharge variables (i.e., duration of treatment (p = 0.08) and regular discharge (p = 0.16)) were no longer significant. The parameter estimate for premorbid %EBW was inversely correlated with the resumption of menses compared with %EBW at the 12-month follow-up, i.e., a high premorbid %EBW lowered the odds of resumption of menses. Using absolute BMI or BMI-SDS instead of %EBW (for all time points) yielded almost identical results.

## Discussion

In the present study, we investigated the resumption of menses 12 months after the initiation of hospital treatment in a large sample of female patients with first-onset AN. In 47% of the adolescent patients, menses spontaneously returned within the first 12 months after admission. This finding corresponds to the findings of previous studies [18,29,30,33]. As expected, deviation from “normal age-appropriate” BMI (%EBW) at the 12-month follow-up had the largest impact on menstrual status. Patients who resumed menses had an average BMI of almost 19 kg/m^2^ (or BMI-SDS: -0.8), whereas those who had not resumed menstruation had, on average, a significantly lower BMI of 17.5 kg/m^2^ (BMI-SDS: -1.4). This result is in accordance with previous studies in adolescent and adult AN patients. Swenne et al. [28] found that adolescent patients had a BMI of 19.2 kg/m^2^ (or BMI-SDS: -0.59) when they started menstruating again. In several studies in adult AN patients, the mean current BMI in those who had resumed menses was found to be between 18.5 and 19.1 kg/m^2^ and was significantly higher than the BMIs of patients with persistent amenorrhea [24-26,29]. In the study of Golden et al. [30], patients had a mean BMI percentile of 27 at resumption of menses. Although the BMI at the exact time point of resumption of menses was not evaluated in our study, we found that those patients who were menstruating at the 12-month follow-up had a similar mean BMI percentile of 24. A recent study examined resumption of menses in relation to %EBW and found that two-thirds of the adolescent patients resumed menses at weights of approximately 95% EBW [33]. Golden et al. [27] also reported that 86% of the patients who achieved approximately 90% of their standard body weight resumed menses within 6 months. In our study, patients resumed menstruation at a very similar %EBW. The mean %EBW of the patients who spontaneously resumed menses was 91 at the 12-month follow-up, while those who were still amenorrheic had weights of approximately 86% EBW. However, in the present study and other studies, the current (age-adjusted) BMIs of amenorrheic and menstruating girls exhibited considerable overlap [22,25,28,30,41,42], which suggests an impact of additional factors on the resumption of menses.

Thus, an important aim of this study was the identification of further anthropometric and clinical factors that may predict the normalization of menstrual status and are easily acquired at the time of initial hospital treatment.

The absence of menarche at hospital admission strongly predicted persistent amenorrhea at the 12-month follow-up. Patients who did not have their menarche before the onset of AN were unlikely to start menstruating within the first 12 months after admission. This important finding has not been reported previously. However, many other studies included only patients with secondary amenorrhea (e.g., [22,25,29,41], while in our study, one-fifth of the patients were still premenarchal at admission. The finding that the absence of menarche before the onset of AN predicted a worse outcome corresponds to the results of other studies that a very low age at onset, e.g., childhood AN, is associated with a worse long-term outcome compared with that in patients with adolescent onset of AN [2,43,44].

The duration of hospital treatment also had predictive value. Patients who required longer hospital stays to reach their target weights (16.8 weeks vs. 14.7 weeks) were more likely to have persistent amenorrhea at the 12-month follow-up. However, the factors underlying this finding remain unclear. Longer hospital treatment durations may imply more severe courses of illness that cause difficulties in gaining weight or considerable differences between the weight at admission and the target weight. In our study, the different variables at discharge, such as %EBW, regular discharge at target weight, and duration of treatment, were strongly related, and their individual effects could not be precisely distinguished. Willer et al. [45] also reported that increased lengths of hospital stays predicted rehospitalization, which may be an indicator of worse outcomes. However, longer hospital treatment durations also shortened the time between discharge (discharge normally occurred when the target weight was maintained for two weeks) and the 12-month follow-up. It has previously been shown that the length of time after weight rehabilitation is an important determinant of the recovery of the menstrual cycle [22] and that some adolescent girls with AN only resume menses several years after weight restoration [21,27]. Thus, for complete recovery of menstrual function, longer periods of weight stabilization without energy deficits may be necessary [23].

The achievement of target weight (defined as the 15-20^th^ age-adjusted BMI percentile) before discharge from hospital treatment was a significant positive predictor of the resumption of menses at the 12-month follow-up. After adjusting for other relevant clinical variables, patients with high premorbid BMIs were found to be less likely to resume menses by the follow-up, most likely because there was still a significant difference between target weight (as defined above) and the weight at which menstruation was suspended. Accordingly, the findings of other studies indicated that premorbid weight [25,26] and the weight at which menstruation ceased may help to predict the weight required for the return of menstruation [27,28]. Thus, the determination of an adequate target weight is essential, as sufficient weight rehabilitation and stabilization are necessary for the resumption of menses. Especially in young patients with AN, weight restoration and recovery of hypothalamic-pituitary-gonadal function are of particular importance, as they are the most important preconditions for bone recovery [13,46]. In addition, sex hormones were shown to be of great importance for brain maturation during adolescence [17,47]. Growth charts based on large population-based data sets can aid in the estimation of appropriate ranges of expected weights based on current age and height. In adolescents with secondary amenorrhea, the weight at which menstruation ceased and premorbid weight should also be considered. Based on our findings and those of others, a target weight between the 15^th^ and 20^th^ BMI percentile seems to be an adequate treatment goal.

### Strengths and limitations

Some limitations of the present study should be considered. First, the time of follow-up was limited to 12 months. Second, the study only included young, non-chronic patients with a relatively short duration of illness and moderate to severe AN. Thus, our results may not be applicable to patients with more chronic courses of AN. Third, biological parameters, such as hormone levels (e.g., gonadotropins, estrogens, and leptin), or data on body composition that may also influence the resumption of menses have not been investigated.

The study also has several methodological advantages. We investigated a large sample of adolescent AN patients who participated in a standardized treatment program and had a low rate of loss to follow-up. The patients in our sample represented a typical population of adolescents with AN seeking inpatient treatment [48-51]. In addition, we were able to assess a rather young sample consisting of a large number of premenarchal patients, which allowed us to demonstrate that early AN onset may result in protracted hormonal deficits. Finally, follow-up data were assessed personally by well-trained and experienced therapists.

## Conclusions

The recovery of menstrual function in adolescent patients with AN should be a major treatment goal to prevent severe long-term somatic and psychiatric sequelae. Patients with premenarchal onset of AN and those with a high premorbid BMI are obviously at particular risk for protracted amenorrhea despite weight rehabilitation. While these two risk factors cannot be influenced by treatment, others can be addressed by adequate treatment strategies. In particular, discharging patients only after they have reached their individual target weight and supporting them to maintain this weight can influence outcome positively. The calculation of an individual target weight should be based on population-based reference data. A target weight corresponding to a range between the 15^th^ and 20^th^ BMI percentile seems to be favorable in terms of the resumption of menses. Whether patients with a higher premorbid BMI may benefit from a higher target weight should be investigated in further studies.

## Competing interest

Dr. Fleischhaker received grants by Bristol-Myers-Squibb, Novartis, and Otsuka; Dr. Bühren, Dr. Dempfle, Dr. Egberts, Dr. Herpertz-Dahlmann, Dr. Pfeiffer, Ms Schwarte, Dr. Timmesfeld and Dr. Wewetzer declare that they have no competing interests for this paper.

## Authors’ contributions

All authors contributed extensively to the work presented in this paper. BH-D was in charge of conception and design of the study and supervised the project. RS, KB, CF, KE, EP and CW carried out treatment of the patients and data acquisition. AD and NT performed the analysis and supported the interpretation of the data. KB and AD wrote the article. BH-D did the revision of the manuscript. All authors read and approved the final manuscript.

## Pre-publication history

The pre-publication history for this paper can be accessed here:

http://www.biomedcentral.com/1471-244X/13/308/prepub
